# Two methodologies for brain signal analysis derived from Freeman Neurodynamics

**DOI:** 10.3389/fnsys.2025.1570231

**Published:** 2025-04-15

**Authors:** Jeffery Jonathan Joshua Davis, Ian J. Kirk, Robert Kozma

**Affiliations:** ^1^MIND Lab, School of Psychology and Centre for Brain Research, University of Auckland, Auckland, New Zealand; ^2^The Embassy of Peace, Whitianga, New Zealand; ^3^Department of Mathematics, University of Memphis, Memphis, TN, United States; ^4^School of Informatics, Obuda University, Budapest, Hungary; ^5^Kozmos Research Lab, Boston, MA, United States

**Keywords:** electrocorticogram, electroencephalogram, Freeman Neurodynamics, meaning, intentional action, Hilbert transform, Fourier transform, pragmatic information

## Abstract

Here, Freeman Neurodynamics is explored to introduce the reader to the challenges of analyzing electrocorticogram or electroencephalogram signals to make sense of two things: (a) how the brain participates in the creation of knowledge and meaning and (b) how to differentiate between cognitive states or modalities in brain dynamics. The first (a) is addressed via a Hilbert transform-based methodology and the second (b) via a Fourier transform methodology. These methodologies, it seems to us, conform with the systems' neuroscience views, models, and signal analysis methods that Walter J. Freeman III used and left for us as his legacy.

## 1 A brief introduction to Freeman Neurodynamics in the study of brains

Since the publication of Walter Freeman's book “Mass Action in The Nervous System” (Freeman, 1975), much water has flowed under the bridge. In the text, Freeman attempted to answer questions like, “What are the neural mechanisms, and what is the behavioral significance of the electroencephalogram (EEG)?” Most of the experimental aspects of his study are based on the mammalian olfactory system. Here, the neuron is depicted in the context of its interrelation with other neurons that form interactive masses. Initially, the neuron is described with the aid of linear differential equations, with some descriptions of “lower level models” that are used to derive models at a “higher level.” The exploration continues with “non-linear input output relations of neurons in masses” formulated as coefficients in linear differential equations that are amplitude dependent, and the effects on the behavior of masses are analyzed. The electrical fields are conceived as the main way to indirectly observe the activity of neurons, individually and in masses, and the properties that emerge from feedback within neural masses are addressed. Finally, a description of the mechanisms of “neural signal processing” at the level of neural masses is provided.

Fifteen years later in “Neurodynamics: An Exploration in Mesoscopic Brain Dynamics” (Freeman, 2000), Freeman focuses on presenting data, models, and experimental techniques with a mesoscopic approach to brain dynamics. To understand the mesoscopic level in brain dynamics, Freeman conveniently invokes the notion of neuropil, as populations of neurons, to move away from the microscopic level, the neuron as the center of attention of the neuron doctrine.

Neuropil is the densely interconnected tissue in the cortex, which is the most complex substance in the known universe (Freeman et al., 2001). Moreover, a neuropil with 10,000 to 100,000 neurons can be taken as the building block of Freeman neurodynamics; the K0 sets (Freeman, 1975) and more complex and larger populations of neurons, described by Freeman as “mature neuropil,” seem to be fundamental to his mesoscopic approach when he wrote in his study (Freeman, 2000) that:

In cellular terms, in a mixed population of excitatory and inhibitory neurons the excitatory cells excite each other as well as the inhibitory cells, and the inhibitory cells inhibit each other as well as the excitatory cells. This configuration of excitatory and inhibitory populations encapsulates this topology of synaptic interactions in mature neuropil, so it is the centerpiece of mesoscopic brain dynamics. It is both the conclusion of microscopic cellular studies and the starting point for modeling mesoscopic interactions.

The macroscopic level is conceived as large brain systems or the whole brain. When studying the mesoscopic level, the focus is placed on the study of brain dynamics as manifested in the electromagnetic fields measured at the cortex or scalp via a network of electrodes used in electrocorticogram (ECoG) or electroencephalogram (EEG) systems of measurement. Then, the signals are analyzed as spatio-temporal patterns of brain dynamics and displayed as brain dynamics movies emphasizing theta, alpha, beta, and gamma rhythms. These models show the outlines of the forms that are likely to be taken by field theories describing the global cooperative interactions governing the functions of entire cerebral hemispheres through the formation of patterns that resemble the dynamics of tornados, turbulence, and fractalness in spatio-temporal brain dynamics, of large-scale neural activity. In that way, the mesoscopic level of interaction serves as a stepping stone to bridge the gap between the neuron and the large systems of the brain, displaying large-scale integration in global brain dynamics and states. This requires many empirical models and experiments accomplished at a mesoscopic level.

In studies conducted by Barrie et al. (1996) and Freeman and Barrie (2000), evidence was found for the self-organization of Amplitude Modulated (AM) patterns via conditioned stimuli, where presumably the primary receiving areas maintained a master–slave interaction. These relevant spatio-temporal AM patterns describing the destabilized areas of the cortex after stimuli were found in gamma activity on the millimeter scale. These AM patterns, together with Instantaneous Frequency (IF) and Analytic Phase (AP) measurements derived from the Hilbert transformed signals in filtered relatively narrow bands, became the object of much study years after the publication of this book till the present time (Freeman and Quiroga, 2013; Kozma and Freeman, 2016).

It is important to mention that Freeman and Quiroga focused on ECoG and EEG brain signals derived from animal and human studies. This was the object of their book “Imaging Brain Function with EEG—Advanced Temporal and Spatial Analysis of Electroencephalographic Signals,” as they wrote:

“*This book is about temporal and spatial patterns that we find in the electric fields on the scalp (electroencephalogram, EEG) and cerebral cortex (electrocorticogram, ECoG) (Lopes da Silva*, *1993**; Basar*, *1998**). The patterns are enigmatic, ephemeral, easily dismissed as noise, and by most accounts epiphenomenal (Freeman and Baird*, *1989**). Yet, some of the patterns are neural correlates of intentional actions, specifically the perception and discrimination of sensory stimuli by alert, aroused human and animal subjects. For this reason, they have become a focus of our experimental and theoretical investigations. What can they tell us about how brains work? What tools do we need to record and analyze them?”* (Freeman and Quiroga, 2013, p. vii, preface).

It is precisely this approach that has been inspirational to Davis's work in collaboration with Robert Kozma and Walter J. Freeman III, until his last days on earth (24 April 2016 was the day of his passing).

In Freeman's last book with Kozma, they presented a set of models and studies of brain dynamics. Freeman's pioneering work included establishing the hierarchy of neurodynamics from K0 to KIII sets in the 70s, starting from granules of 10s or 100s of thousands of neurons to sensory cortices (Freeman, 1975). This work was extended in the early 2000s to intentional neurodynamics through multisensory integration and Gestalt formation in KIV sets (Kozma and Freeman, 2003; Kozma, 2007; Freeman and Erwin, 2008).

This is very much in line with and supportive of our exploration here since, in order to study how the brain participates in the creation of meaning for intentional action, we have focused on oscillations and wave dynamics caused by fields in the brain, as Freeman and Kozma have suggested, to complete the neuron doctrine in their book titled “Cognitive Phase Transitions in the Cerebral Cortex—Enhancing the Neuron Doctrine by Modeling Neural Fields” (Kozma and Freeman, 2016). It is important to note that they have dedicated their book to us “[…] scientists who persevere in questioning prevailing dogma in search of wisdom in the frontiers of neuroscience” (p. vi), and a set of scholars have expressed their commentaries about the value of this work in their own field of research. In the following, we mention some of the main ideas of some commentary (chapters) from some scholars.

We start with Bernard J. Baars, who states that “WJ Freeman and Robert Kozma have developed a strikingly novel approach to mass action in the brain, especially the horizontal dendritic neuropil of cortex.” Moreover, in his commentary, he “considers selected hypotheses from Kozma and Freeman (in press) from the viewpoint of Dynamic Global Workspace Theory, a rigorous effort to account for conscious (reportable) brain events.” (Baars, 2016). Steven L. Bressler indicates that the purpose of his commentary “ […] is to advance our understanding of the functional actions that occur between different areas of the mammalian neocortex.” Then, he emphasizes that the “[…] topic is of immense importance to the question of the neural basis of cognition, both in animals and humans.” (Bressler, 2016). Frank W. Ohl reviews “a set of studies that have recently improved our understanding of the nature of large-scale coordinated activity on a mesoscopic scale […] by exploiting two experimental conditions.” (Ohl, 2016). Hans Liljenström explores “both upward and downward causation in cortical neural systems, using computational methods with focus on cortical fluctuations.” He highlights the development of models of “paleo- and neocortical structures, in order to study their mesoscopic neurodynamics, as a link between the microscopic neuronal and macroscopic mental events and processes.” (Liljenström, 2016). Paul J. Werbos has provided constructive criticism contrasting his views with the ones of Kozma and Freeman. He has remained open to the possibility that field effects could be important to studying and understanding the brain and mind and given us a plausible quantum perspective (Werbos, 2016).

Finally, we provide the reader with a set of quotes relevant to our next section, summarizing the main ideas of the approach taken by Vitiello (2016), as follows:

1. “The observed dynamic amplitude modulated (AM) assemblies of coherently oscillating neurons are described in the frame of the quantum field theory of spontaneously broken symmetry theories.”
2. “In the process of formation of the coherent AM patterns, the brain goes from disordered, gas-like, high entropy regime to liquidlike organized neuronal configurations of low entropy, and a representation in terms of thermodynamic generalized Carnot-Rankine cycles is proposed.”
3. “The resulting thermodynamic model incorporates criticality and phase transitions. Long range correlations at the basic quantum level may sustain emphasis [sic] in neuronal interactions generating AM patterns.”
4. “The formation of topologically non-trivial structures, such as vortices, null spykes, phase cones observed in a criticality regime is described in the frame of the dissipative model.”

The methodologies to be explored in the following sections and some results obtained with their application have been grounded on the study by Freeman and Kozma. Here, the focus, as stated before, is on answering two questions via these methodologies: (a) how the brain participates in creating knowledge and meaning and (b) how to differentiate between cognitive states or modalities in brain dynamics. It is important to note that EEG and ECoG signal analysis could be complemented with other techniques, such as functional magnetic resonance imaging, that could provide a more robust and complementary spatial assessment of brain activity (Eichele et al., 2005, 2008; Freeman et al., 2009; Bressler and Menon, 2010; Freeman and Quiroga, 2013). Other relevant approaches have been considered by Werbos and Davis (2017), as well as studies on fuzzy systems applied to EEG (Yu et al., 2025; Cao et al., 2020), are beyond the scope of this study.

Of the two methodologies we present here, one methodology is centered around the Hilbert transform and the other is centered around the Fourier transform (Kamen and Heck, 1997; Hsu, 1995) via the computation of the power spectrum derived from the Fast Fourier transform algorithm or the periodogram. Before explaining the methodologies, let us delve into one of the central ideas in Freeman's Neurodynamics, the ideas of meaning and intentionality.

## 2 Meaning and knowledge creation for intentional action

Freeman's Neurodynamics presents a conception of the brain that is very different from a computer, a processor of information only. As a great system neuroscientist, he described the brain as a living organ within a living body, engaged with its environment, geared to the creation of knowledge, and meaning for intentional action. He told us that the currency of the brain, far from being meaningless sensory information only, happens to be meaning, comprising various categories of meaning, as they inform intentional action in the world, shared with fellow human beings, animals, plants, and rocks, among other elements of nature and social life. He gave us a very thorough account of this pioneering idea in his work (Freeman, 2000, 2003, 2004b).

He managed to inspire scientists and philosophers alike to think deeply and formulate testable theories about how the brain derives meaning from sensory input, after large-scale integration in the form of Gestalts, that lead to the execution of plans of action. A plausible mathematical formulation of intentional neurodynamics is given in the KIV model (Kozma and Freeman, 2003), a culmination of decades-long work on the hierarchy of Freeman K sets (Freeman, 1975).

**Figure 1:**
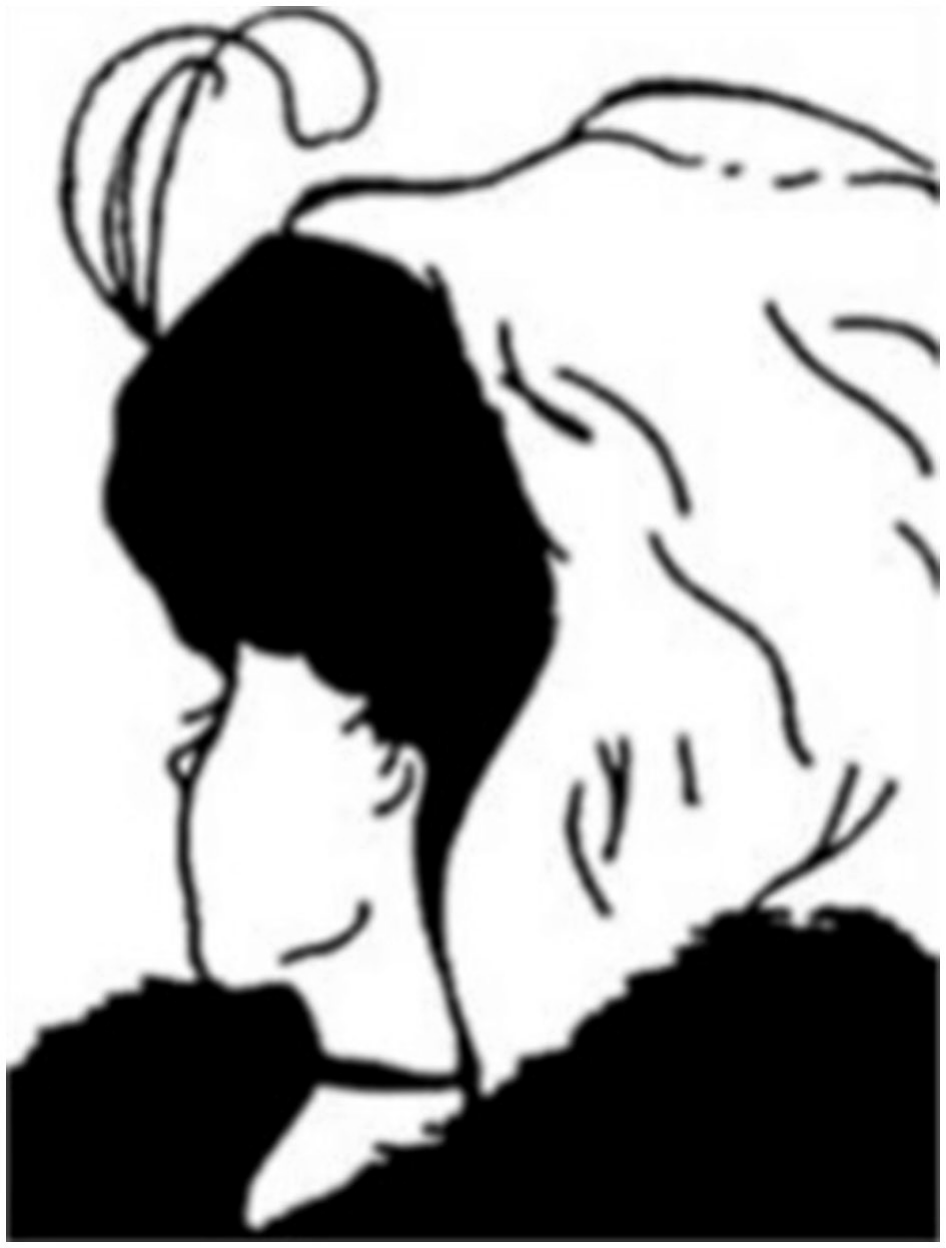
Is used here in an experiment for the reader in meaning creation in brain dynamics. Credit: “My Wife and My Mother-in-Law,” William Ely Hill, Puck, Vol. 78, No. 2018 (November 6, 1915).

The reader is invited to experience how meaning emerges when gazing at the image in Figure 1 and to then write down, from the start of the experiment, the times at which meaningful images emerge, at least two of them. This will give the reader a notion of the time it takes for meaning to arise, considering that he or she has been gazing at meaningless sensory information for a while until meaning emerged. It is suggested that the reader take the time now to do the experiment and then continue reading!

If the reader stared long enough at Figure 1, he or she may have witnessed the brain creating two meaningful images, an old lady and a young woman, both in the same picture. This is remarkable, as one or both images could have gone unnoticed until and unless the brain “did the trick” to show them to the reader's visual field of perception.

Some questions then are as follows: What would be the AM patterns associated with this gazing task while the reader waits for the meanings to reveal themselves? What would the brain be doing at the “eureka” moment? What kind of activity would the brain manifest while writing the times (intentional actions) at which the images happened? What kind of transitions would be observed in brain dynamics from one brain state to the other?

These questions have been addressed in several studies where intentionality has been grounded in the philosophy of Aquinas, concerning intention and brain dynamics (Freeman, 2008). Further work was done by others in studies of the hypothesized Cycle of Creation of Knowledge and Meaning (CKM), from basic animal studies to human studies associated with meditative states and transcendence (Davis et al., 2015a,b; Davis and Gillett, 2023; Davis et al., 2024). Freeman and Kozma gave us a set of models and studies of brain dynamics that are very much in line with and supportive of our exploration here (Freeman et al., 2001; Kozma and Freeman, 2003, 2016).

The next section describes the signal processing methodologies that allow for the classification of cognitive states from different perspectives.

On one hand, we can study the CKM in different brain modalities, such as meditation (MED) and scrambled word resolution (WORDS). We can do that for each frequency band selected and then understand the similarities and differences between participants in different modalities for different bands. In these kinds of studies, we measure pragmatic information peaks and the number of peaks per second computed from the analytic signals derived from the Hilbert transform.

On the other hand, the distinction between or classification of different cognitive states can also be derived from applying robust comparative statistical analysis to measures such as the Shannon entropy index (Shannon and Weaver, 1971), the Pearson's first order skewness coefficient (Pearson, 1895), the dominant frequency, and power associated measures, derived from the normalized power spectrum.

## 3 Hilbert and Fourier transform-based brain signal analysis methodologies

The Hilbert Transform Methodology (HTM) has been used by Davis et al. (2024), and the Fourier Transform Methodology (FTM) has been applied by Davis et al. (2023a). Here, both methodologies are described, with the aim that the reader who is interested in applying them to his or her own studies will have a good guiding document to do so. In Table 1, a summary of the steps involved in each methodology is presented.

**Table 1:** Description of the steps for each methodology used for brain signal analysis.

**Steps HTM**	**Comments**	**Steps FTM**	**Comments**
Remove Artifacts from raw EEG data for each electrode to obtain clean data.	Use a Notch filter to remove 50–60 Hz. Filter all types of artifacts and detrend the data.	Remove Artifacts from raw EEG data for each electrode to obtain clean data.	Use a Notch filter to remove 50–60 Hz. Filter all types of artifacts and detrend the data.
Filter the Clean EEG data in the band of interest for each electrode.	Use an FIR bandpass filter with appropriate parameters. The signals ought to be ideally sampled at 1,000 Hz.	Compute the Power Spectrum using the Fast Fourier Transform or the Periodogram, for each electrode, in sub-windows of 500 ms.	Normalize each Power Spectrum in each sub-window, for each electrode, and save them for further computations.
Apply Hilbert Transform.	Compute Analytic Amplitude (AA), Analytic Phase (AP), and Instantaneous Frequency (IF) for all electrodes.	Compute the Shannon Entropy Index (H) from the Normalized Power Spectrum for each electrode in each sub-window.	You will need the probability per frequency band derived from each Normalized Power spectrum.
Compute Pragmatic Information Index (PI) and Number of Peaks per Second (NPS).	PI= AA^2^*/*ED, where ED is the Euclidean distance between the AA or AP signal in each particular time step t^*^, from all electrodes.	Find the dominant frequency (the mode) for each power spectrum.	The Normalized Power Spectrum is treated as an empirical probability distribution.
		Compute Pearson's 1^st^-order skewness coefficient (PSk) from the Normalized Power Spectrum for each electrode in each sub-window.	Compute the mode, mean, and standard deviation of the empirical probability distribution derived from the Normalized Power spectrum.
		Compute Total Power (TP) from the Normalized Power Spectrum for each electrode in each sub-window.	Compute mean and standard deviation for H, PSk, DF and TP for each electrode over all sub windows. This can also be done per participant, modality and band according to the needs for analysis.

Software, such as Octave, Python, or MATLAB, among others, will be needed to perform all computations.

Fourier transform-based frequency analysis has been used in many relevant EEG studies when stationarity conditions are met or achieved when dealt with properly to compute the power spectrum. However, EEG signals are noisy, non-stationary, non-linear, showing discontinuities associated with sometimes abrupt transitions from brain to brain state (Huang et al., 1998). In the case of rapid changes and transients of brain signals, Hilbert's analysis showed clear benefits since the end of the 20th century (Le Van Quyen et al., 2001). The Hilbert transform is a linear operator that allows for the analysis of non-stationary signals via the study of the instantaneous frequency that derives from the Analytic Phase (Freeman and Quiroga, 2013).

The mathematical techniques associated with these methodologies are extensively described by Freeman and Quiroga (2013), along with sound neurophysiological interpretations. Freeman and Quiroga have made a great effort to explain the benefits of applying the Hilbert transform when analyzing EEG and ECoG signals. They also explain how to interpret changes in analytic amplitude and phase of oscillations in different frequency bands, as interpreted by systems and cognitive neuroscientists, such as Buzsáki (2006). For detailed explanations, see Chapters 2, 4, 6, 9, and 10 of the study by Freeman and Quiroga (2013). In general, the Analytic Phase and Instantaneous Frequency allow identifying synchronization and desynchronization moments between signals as measured by different electrodes. Analytic amplitude is a good marker, showing changes related to cognitive tasks, for example, particularly in the Gamma frequency band (Kozma and Freeman, 2017; Buzsaki, 2019). Concerning the Pragmatic Information index derived from Analytic Amplitude, it has the property of being an excellent marker of the onset of changes in amplitude until the brain returns to background activity after relevant stimuli are detected and processed (Freeman, 2004a, 2005; Freeman and Vitiello, 2006).

Alternative methods to the Fourier transform have been proposed, namely, time-varying methods such as the short-time Fourier transform, the use of Wavelets, and the Hilbert transform. It is important to note that using Morlet wavelets and the Hilbert transform has also shown benefits when dealing with complex, non-stationary, and non-linear signals (Quiroga et al., 2002; Freeman and Quiroga, 2013). A detailed description of each methodology is presented with a subset of the data obtained from a study in human cognition, where EEG experiments were conducted in six different modalities^1^ for 20 participants. The data were collected at Ian J. Kirk's Lab, Center for Brain Research at The University of Auckland in New Zealand. The results are shown in a set of publications by Davis et al. (2023a,b).

## 4 Hilbert transform-based brain signal analysis methodology

To derive signals in different bands and to be able to learn about the dynamics of meaning creation, we had to filter the signals and then perform a Hilbert transform as described in Equations 1, 4 (King, 2009; Hahn, 1995).

By applying the Hilbert Transform, each EEG electrode signal s(t) is transformed to **SA**(t) as follows:


(1) $$\begin{array}{l} \text{S}\text{A}\left(t\right)\;=\;s\left(t\right)\;+\;i\;{s\left(t\right)}^{*} \end{array}$$

(2) $$\begin{array}{l} \text{S}\text{A}\left(t\right)=\text{A}\text{A}\left(t\right)e^{i\;\text{A}\text{P}\left(t\right)} \end{array}$$

where s(t)^*^ = $\frac{1}{\pi }$ p.v. $\int_{-\infty }^{+\infty }\frac{s\left(t^{\prime }\right)}{\left(t-t^{\prime }\right)}dt^{\prime }$ and where p.v. is the Cauchy Principal value.

We compute the Analytic Amplitude (AA), Analytic Phase (AP), and Instantaneous Frequency (IF) as follows:


(3) $$\begin{array}{l} \text{A}\text{A}\left(t\right)=\sqrt{{s\left(t\right)}^{2}+{{s\left(t\right)}^{*}}^{2}}\;;\;\text{A}\text{P}\left(t\right)=\mathrm{atan}\left(\frac{{s\left(t\right)}^{*}}{s\left(t\right)}\right) \end{array}$$

(4) $$IF\left(t\right)=\left(\frac{1}{2\pi }\right)\left(\frac{\Delta AP\left(t\right)}{\Delta t}\right)=\;\left(\frac{1}{2\pi }\right)\left(\frac{AP\left(t\right)-AP(t-\Delta t}{\Delta t}\right)$$

The MATLAB “hilbert” function was used to compute the imaginary part s(t)^*^ from the real-valued signal s(t).

**Figure 2:**
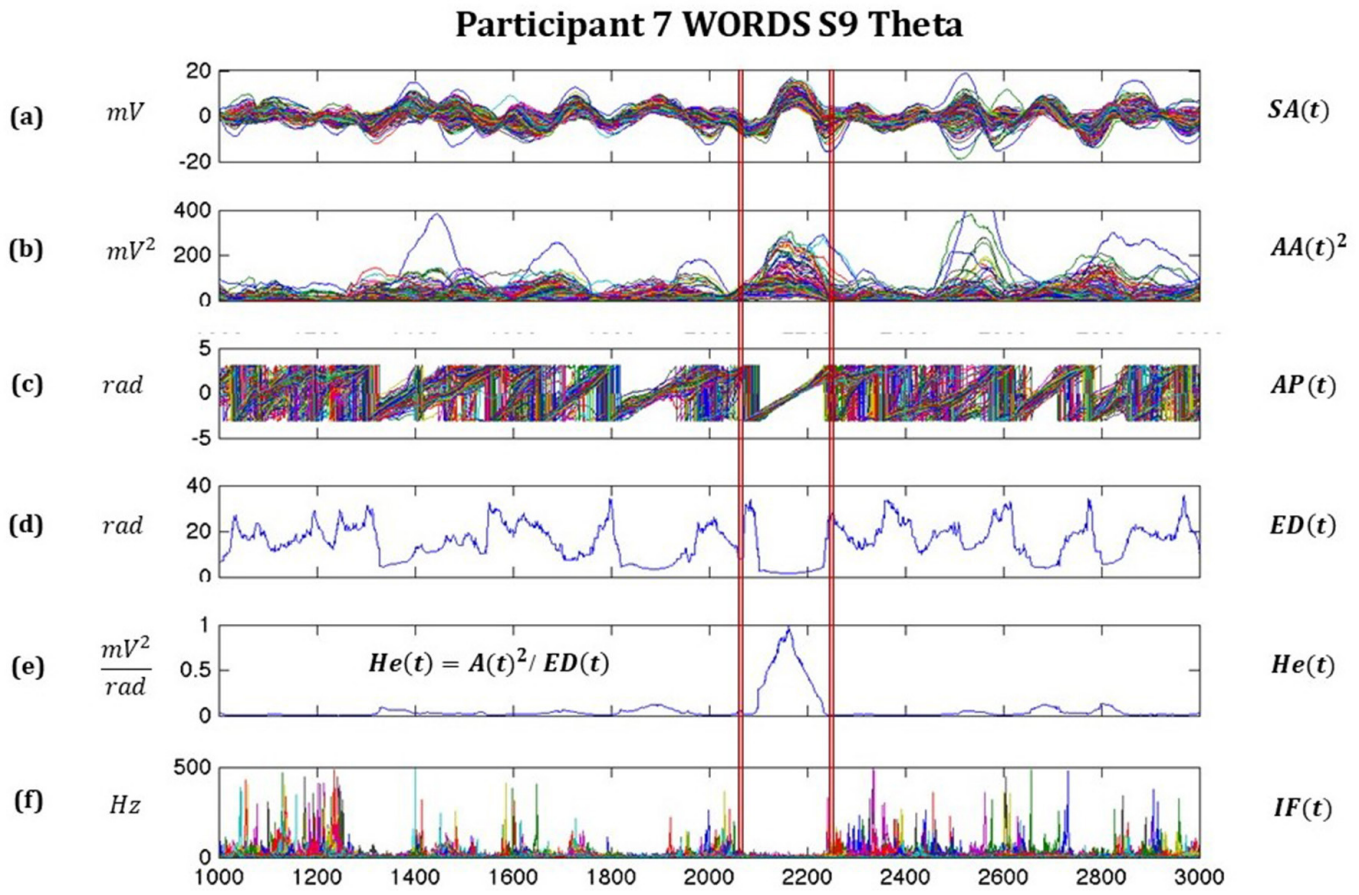
Illustrates moments of synchronizations and desynchronization for all 128 channels, with highly synchronized signals between around 2075–2250 ms, where **(a)** is the signal amplitude SA(t), **(b)** the analytic amplitude squared AA(t)^2^, **(c)** Analytic Phase AP(t), **(d)** Euclidean distance ED(t) based on AP(t), **(e)** pragmatic information index He(t), and **(f)** instantaneous frequency IF(t).

In Figure 2, we observe some of the important features of the signals derived from the Hilbert transform and how they relate to each other so that the moments of synchronization and desynchronization for all 128 channels are easily identified. A very relevant moment where we observe highly synchronized signals is from 2,075 to 2,250 ms, where the signal amplitude SA(t), the analytic amplitude squared AA(t)^2^, the Analytic Phase AP(t), the Euclidean distance ED(t) based on AP(t), the pragmatic information index He(t), and the instantaneous frequency IF(t) are shown.

Relevant studies show the use of this methodology throughout the years (Freeman et al., 2003; Kozma et al., 2012; Davis et al., 2015a,b; Kozma and Freeman, 2003, 2016).

A comparison between two participants, based on the pragmatic information index in the modalities MED and WORDS, is presented.

### 4.1 Computation of the pragmatic information index

To analyze the origin, structure, and role of background EEG activity, Freeman invoked the notion of Pragmatic Information (PI, H_e_ index) in Freeman (2004a), where he writes that:

This ratio is analogous in form to a definition of order by Haken (1983, p. 181, Equation 6, p. 178), and to “pragmatic information” defined by Atmanspacher and Scheingraber (1990) using the concept of “efficiency” (Equations 8–10) as a “fundamental extension of Shannonian information” (pp. 731–2). Thus, He might be regarded as an index of the time-varying quantity of the information in wave packets that is displayed in sequences of their AM patterns.

Freeman (2005) writes that:

Recent advances in the application of the Hilbert transform to EEGs in the beta and gamma ranges (Freeman and Rogers, 2002; Freeman, 2004a) led to the detection in the EEG of spatial AM patterns having high degrees of coherence, stability, and intensity. These epochs were identified with high values of an index, He, that Atmanspacher and Scheingraber (1990) labeled “pragmatic information.”

The Pragmatic Information Index (PI, H_e_) has been used to detect the creation of knowledge and meaning in brain dynamics in a way that would allow us to explain plans of action after a salient stimulus has been identified as meaningful.

In the study by Freeman and Vitiello (2006), they state that the best predictor of the “*onset times of ordered AM patterns was the ratio of the rate of free energy dissipation to the rate of change in the order parameter.”* The ratio is the pragmatic information index^2^ (PI), *H*_*e*_(*t*), following Atmanspacher and Scheingraber (1990), as a fundamental extension of and alternative to the Shannonian information, and formulated as stated before. In the study by Freeman and Vitiello (2006), they used the approximation given in Equations 8, 9, 10 in Freeman (2004a), as follows:


(5) $$\begin{array}{l} H_{e}\left(t\right)=\langle {\text{A}}^{2}\left(t\right)\rangle D_{e}\left(t\right) \end{array}$$

Let us define Ne as the number of electrodes placed on the scalp of the participants to measure brain activity to derive the squared analytic amplitude as **A**^2^(t), represented as a spatial vector at each time step t, where **A**(t) = {A(t)_1_, A(t)_2_, ..., A(t)_Nc_}, where **A**^2^(t) is treated as an order parameter. The Euclidean distance then is defined as:


(6) $$\begin{array}{l} De\left(t\right)\;=\;{\left|{\text{A}}^{2}\left(t\right)\;-\;{\text{A}}^{2}\left(t\;-\;1\right)\right|}^{2} \end{array}$$

If we choose to express D_e_(t) as a function of ***AP**(t)*, then the computations are as follows in Equations 7–11.

Let us define a time index as t = 1, 2, ….., t^*^,…., T and an electrode index as i = 1, 2, .…, Ne. Then, it follows that:


(7) $$\Delta \;AP{\left(t*\right)}_{i}=AP{\left(t*\right)}_{i}-AP{\left(t*\right)}_{i-1}\forall \;i=2,\;\;Ne,\;\;for\;t=t*$$

(8) $$\Delta \;AP(t*)=\left\{\Delta AP{\left(t*\right)}_{1},\Delta \;AP{\left(t*\right)}_{2},\ldots \Delta AP{\left(t*\right)}_{Ne}\right\}\;$$

(9) $$D_{e}\left(t*\right)={\left|\Delta \;AP(t*)\right|}_{2}\;\forall \;i=2,\;Ne\;$$

(10) $$D_{e}t*=\sqrt{{\sum_{i=2}^{Ne}\left[\Delta \;AP{\left(t*\right)}_{i}\right]}^{2}}$$

(11) $$D_{e}\left(t\right)=\left(D_{e}\left(1\right),\;D_{e}\left(2\right),\;\ldots .,\;D_{e}\left(t*\right)\;\ldots \;D_{e}\left(T\right)\;\right)\forall \;t\;=\;1,\;T$$

where *i* is a particular channel, t^*^ is a particular point in time, *Ne* is the total number of channels (electrodes), and T is the time length of the signal or last temporal point.

**Figure 3:**
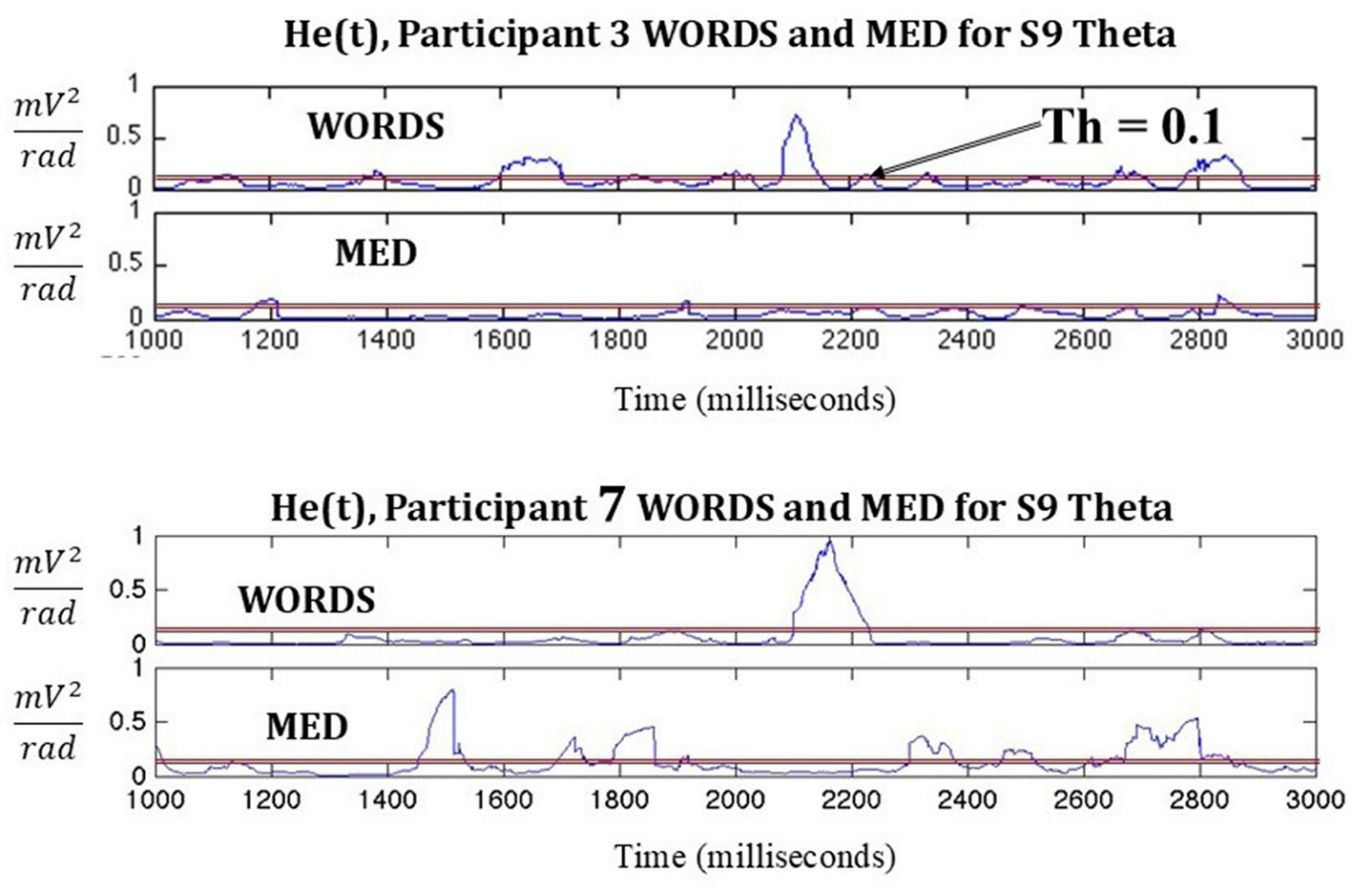
Shows a comparison between two participants in the modalities MED and WORDS in terms of PPS. A threshold (Th) is set to indicate a significant PPS of interest.

Following in Figure 3, a comparison based on He(t) between two participants in the modalities MED and WORDS is shown. It can be observed that Participant 3 shows a smaller number of significant peaks per second (PPS) for the modality MED than for the modality WORDS. For Participant 7, the opposite is observed; the modality MED shows more PPS than the modality WORDS.

**Figure 4:**
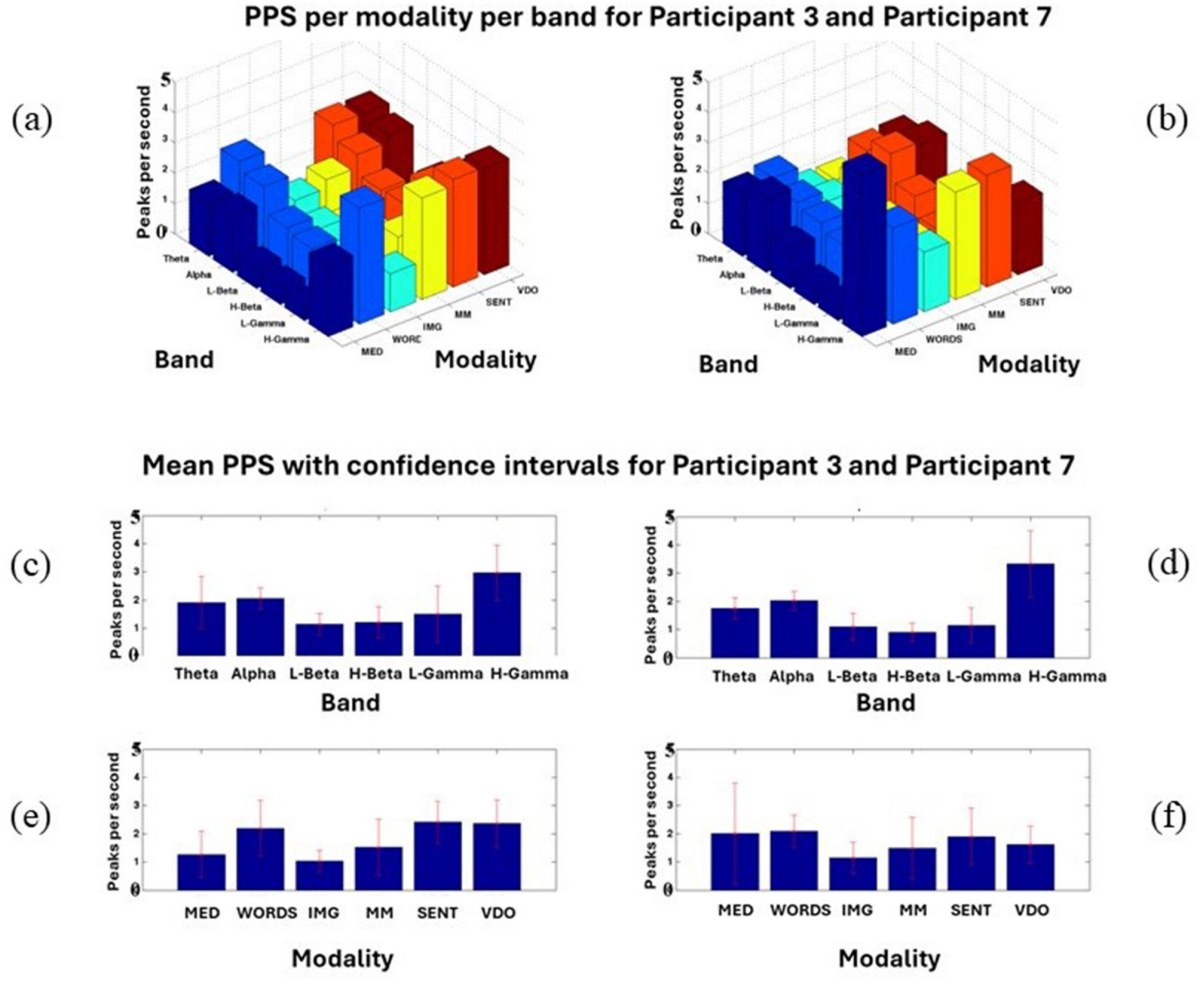
Shows Participant 3 **(a, c, e)** and Participant 7 **(b, d, f)** PPS for every modality and band **(a, b)**, Mean PPS with confidence intervals per frequency band **(c, d)**, Mean PPS with confidence intervals for each modality **(e, f)**. In this figure, all the modalities and bands studied in Davis et al. (2024) are displayed for an illustration only, for Participants 3 and 7.

It must be noted that this is just an example where 2,000 ms of data from stimulus nine (S9) in each modality for the frequency band theta is shown. When taken together, with all the stimuli per modality per participant in each band, some important patterns start to emerge, as shown in Figure 4. Most of the band's behaviors are very similar for all modalities for both participants, as shown in Figures 4a, b.

However, some differences can also be outlined, where Participant 3 shows a significantly lower amount of PPS in the modality MED for the High Gamma (H-Gamma) frequency band than Participant 7, who displays a very high number of PPS for the same modality and band. In the modality WORDS, we observe, in general, higher PPS values for Participant 3 than for Participant 7.

A better assessment can be done in Figures 4c, d, where it can be observed that the modality MED shows less PPS activity for Participant 3 than Participant 7. All bands' activities are shown in Figures 4e, f. Apart from that, most modalities seem to behave statistically *similarly*. This will need further research to be conclusive. It is important to note that in Figure 4, all the modalities and bands studied in the work by Davis et al. (2024) are displayed for illustration only for Participants 3 and 7. From the end of this section onwards, the focus will continue to be on modalities MED and WORDS.

Let us remember that the results presented here rely on twenty (20) participants only, and the modalities selected aim to illustrate the application of the methodology presented. It is important to highlight that this will allow for the exploration of how the brain participates in creating knowledge and meaning, as studied by Davis and Kozma (2013) and Davis et al. (2013). This subject will be elaborated on in the coming section.

Now, we are ready to proceed to the exploration of the power spectrum-based methodology.

## 5 Fourier transform-based brain signal analysis methodology

In this section, several power-spectrum-based indices of interest are introduced to aim at a classification of different cognitive states (Davis et al., 2020). The indices of interest are the Shannon entropy index (H), Pearson's 1st-order skewness coefficient (PSk), Total Power (TP), and Dominant Frequency Band (DF).

The entropy measure H, as introduced by Shannon and Weaver (1971), is used, giving the degree of randomness in an EEG signal, the Pearson's 1^st^-order skewness coefficient (PSk), as a measure of structure described by the degree of asymmetry of a distribution, as shown by Pearson (1895) and further explored by Groeneveld and Meeden (1984). The Pearson's 1^st^-order skewness coefficient, described and formulated by Weisstein (2022), is based on the mean, standard deviation, and mode. Following the computation of H, PSk, TP, and DFB^3^ are shown in detail.

The power spectrum becomes a very useful tool, as explained by Freeman and Quiroga (2013) in Chapter 2. Moreover, in more recent studies, the use of the power spectrum has been proposed to compute the Shannon Entropy Index (H), Pearson's first skewness coefficient (PSk), Total Power (TP), and Dominant Frequency (DF; Davis et al., 2020).

### 5.1 Computation of the H, PSk, TP, and DFB

In this section, the equations for the computations of the measures of interest based on the discrete form of the Temporal Power Spectrum (PSD_t_) are presented as follows:


(12) $$\begin{array}{l} PSD_{t}≐\;PW_{i}\;\left(FB_{i}\right)\;\forall \;i,\;for\;each\;time\;window\;t \end{array}$$

where *PW*_i_ corresponds to the power of frequency band “i” (*FB*_*i*_).

and DFB = max (*FB*_*i*_) ∀ i, for each time window t

or alternatively, the mode of the *PSD*_*t*_

From the normalized PSD_t_, H is derived as follows:


(13) $$\begin{array}{l} H=-\sum_{i=1}^{n}p_{i}*\log_{2}\left(p_{i}\right)\\ \;{where\;p}_{i}=\;\frac{PW_{i}}{TP} \end{array}$$

and TP is the total power computed as:


(14) $$\begin{array}{l} TP=\;\sum_{i=1}^{n}PW_{i} \end{array}$$

The PSk computation is as follows:


(15) $$\begin{array}{l} PSk=\;|\frac{\left(Mean_{PSD_{t}}-Mode_{PSD_{t}}\right)}{SD_{PSD_{t}}}| \end{array}$$

In Equation 15, *Mode*_*PS*_*D*__*t*__ refers to the dominant frequency band (DFB or DF). The parameters *Mean*_*PS*_*D*__*t*__ and *SD*_*PS*_*D*__*t*__ refer to the mean and standard deviation, respectively, derived from the normalized PSD_t_ and treated as a probability distribution.

When computing H, the probability p_i_ derived from the PSD_t_ is used, where the number of a particular band is described by a fixed number “i” for all PSD_t_ when applied to any participant and modality, as follows:


(16) $$\begin{array}{l} PSDt≐\;PW_{i}\;\left(FB_{i}\right)\;\forall \;i=4,\;5,\;6,\;\ldots ,\;48\;Hz \end{array}$$

The need arises sometimes to use a modified version of H called **H**_**c**_, as treated by Rajaram et al. (2017). **H**_**c**_ may apply when comparing brain dynamics for different brain areas in different bands, participants, and modalities, which would depend on a unique and specific probability distribution. This is outside of the scope of the present study.

**Figure 5:**
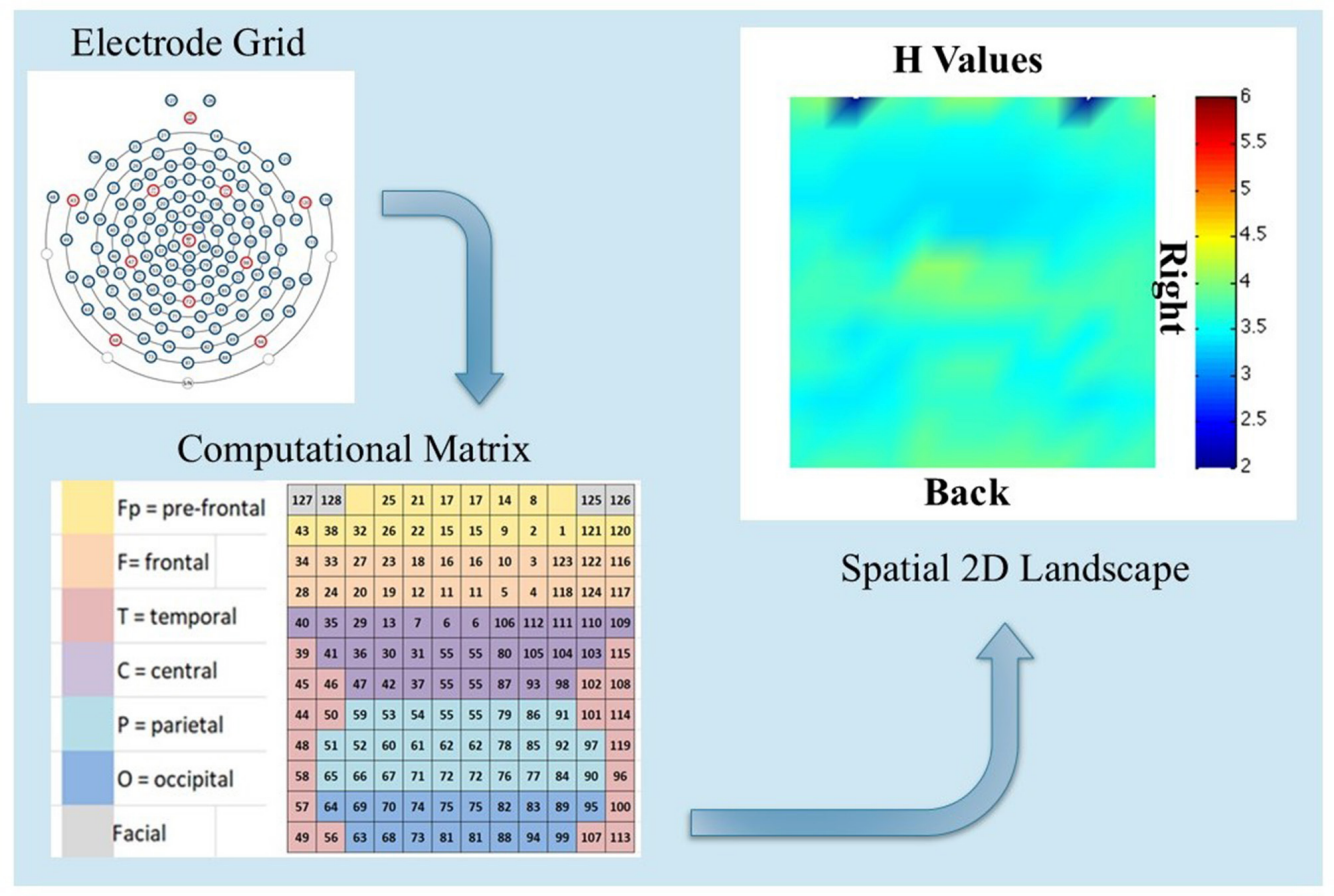
Shows the EEG electrode grid positions with numbers **(top left)**, the computational matrix associated with the grid of electrodes **(bottom left)**, and a spatial 2D landscape image displaying the H measure per electrode, based on the computational matrix **(top right)**. This image could be derived from values of H, PSk or any other measure of interest.

A qualitative comparison between Participants 3 and 7 in the modalities MED and WORDS for H and PSk values is presented. Before doing that, an explanation is given of the basics from where the images for analysis are derived. Figure 5 shows a flow chart to understand the spatial 2D landscape images, displaying values for H to compare different cognitive states between participants in different modalities. This also applies to PSk or any other measure.

**Figure 6:**
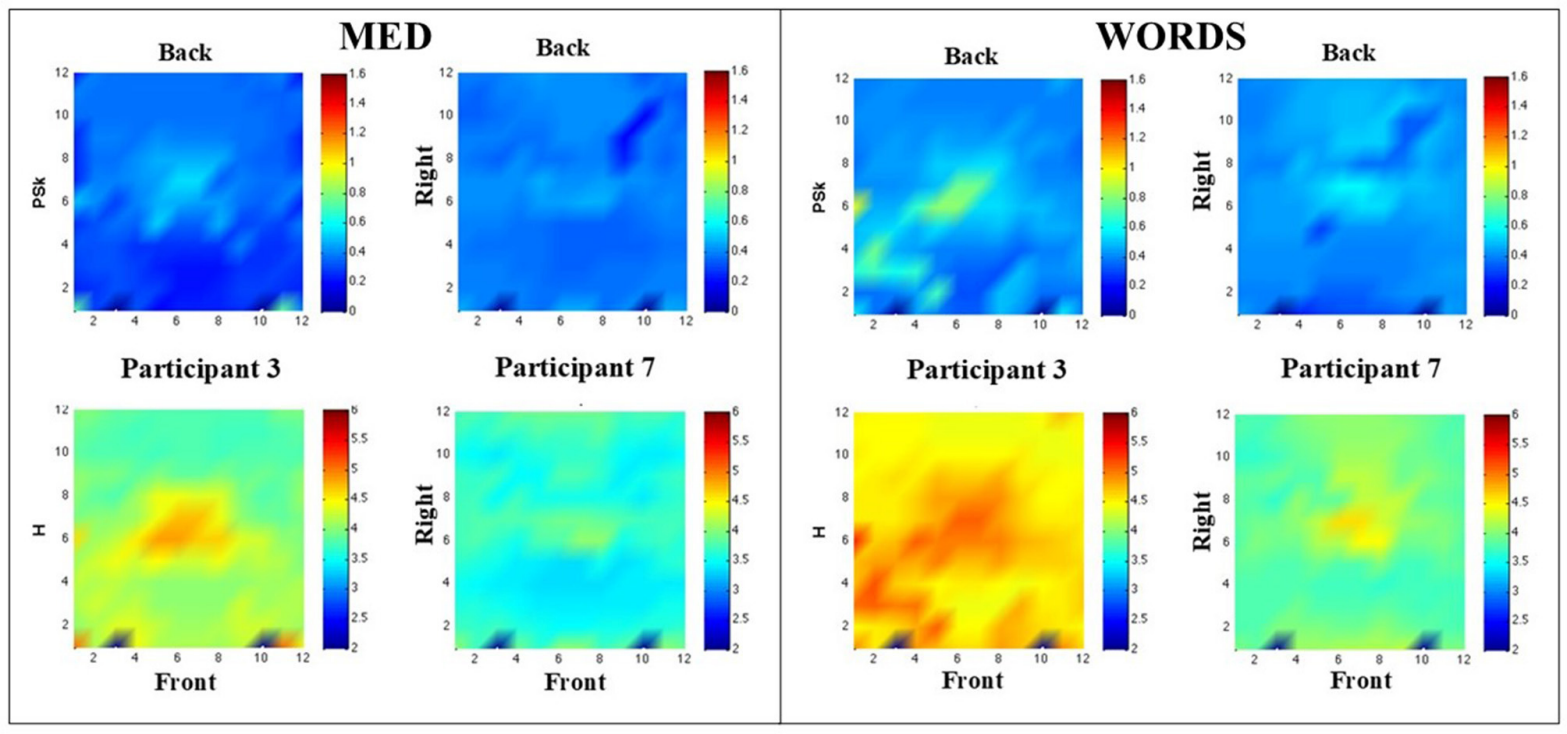
**(Left)** Shows PSk and H values in comparative landscapes, for Participants 3 and 7 in modality MED. **(Right)** Shows PSk and H values in comparative landscapes, for Participants 3 and 7, in modality WORDS. Note that the label Front refers to the front of the brain and similarly for the Back and Right labels.

In Figure 6, some results for Participants 3 and 7 are shown by displaying the H and PSk measures in modalities MED and WORDS.

Particularly in MED (Left), it can be observed that Participant 3 presents lower PSk values in the central prefrontal cortex and higher ones in the center of the central region. Moreover, Participant 7 shows a more even distribution of PSk values for all areas. The entropy values show a similar pattern.

WORDS (Right) shows that the values for PSk are very similar for both participants apart from the central and left and right prefrontal areas, which show higher values of PSk for Participant 3.

In general, the values for H are higher for Participant 3 than for Participant 7, and it can be observed that Participant 3 shows high entropy values for the central, left, and right prefrontal areas.

**Figure 7:**
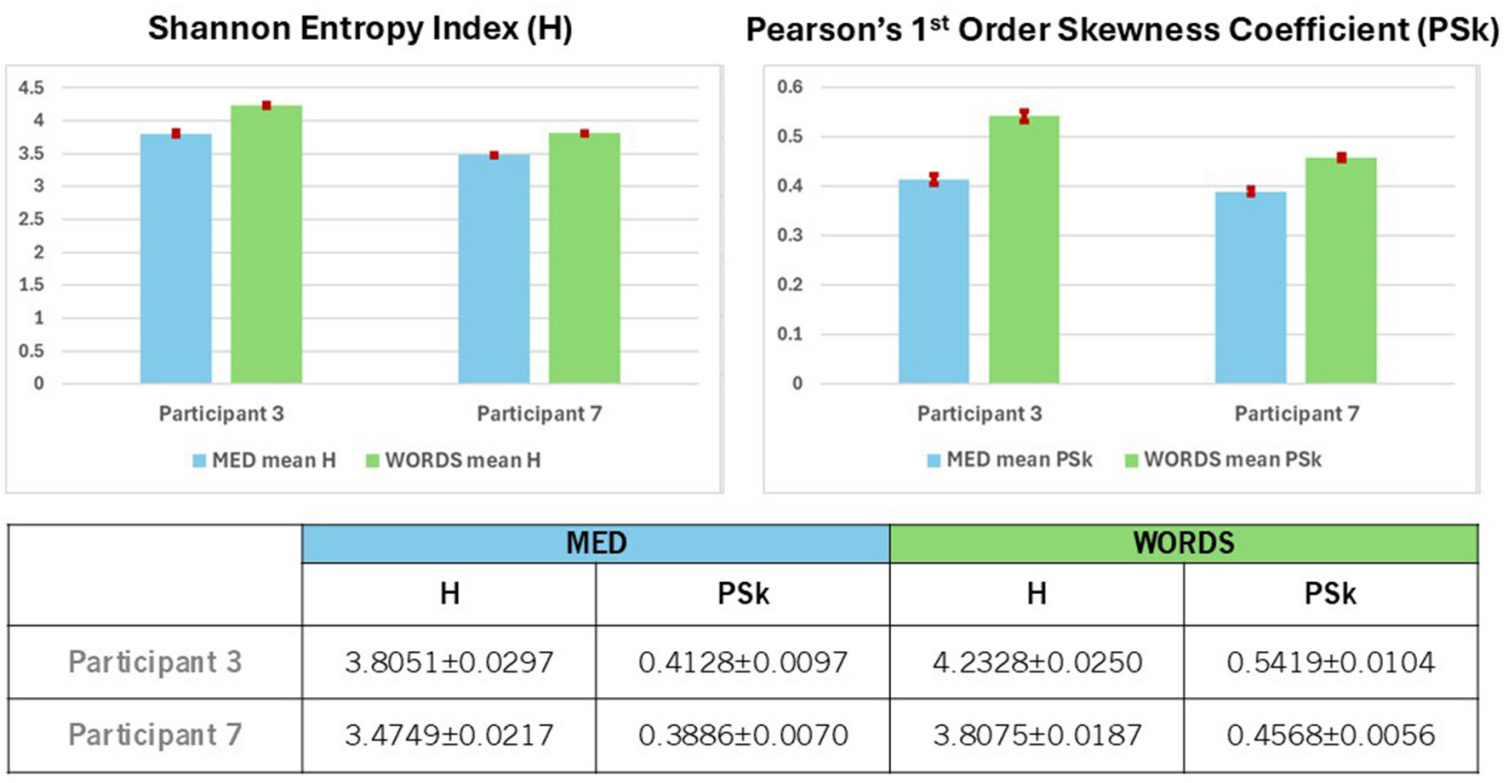
**(Top)** Shows a comparison between Participants 3 and 7 for mean values of H and PSk, with 95% confidence intervals, for modalities MED and WORDS. The mean values are computed from the values of all the electrodes (the whole brain) taken from all 500 ms windows. **(Bottom)** Shows mean values of H and PSk with 95% confidence intervals for modalities MED and WORDS for Participants 3 and 7.

Finally, it is very clear from Figure 7 (Top and Bottom) that both the values of H and PSk are higher for modality WORDS than for modality MED for both participants. It is important to mention that the values for all electrodes (the whole brain) from all 500 ms windows are included in the computation of the mean and errors for H and PSk for both participants in both modalities.

Now, we are ready to move to the Dominant Frequency Band (DFB) analysis via several types of images and displays. The first type shows the time spent in the DFB in different brain areas and, overall, in the whole brain.

**Figure 8:**
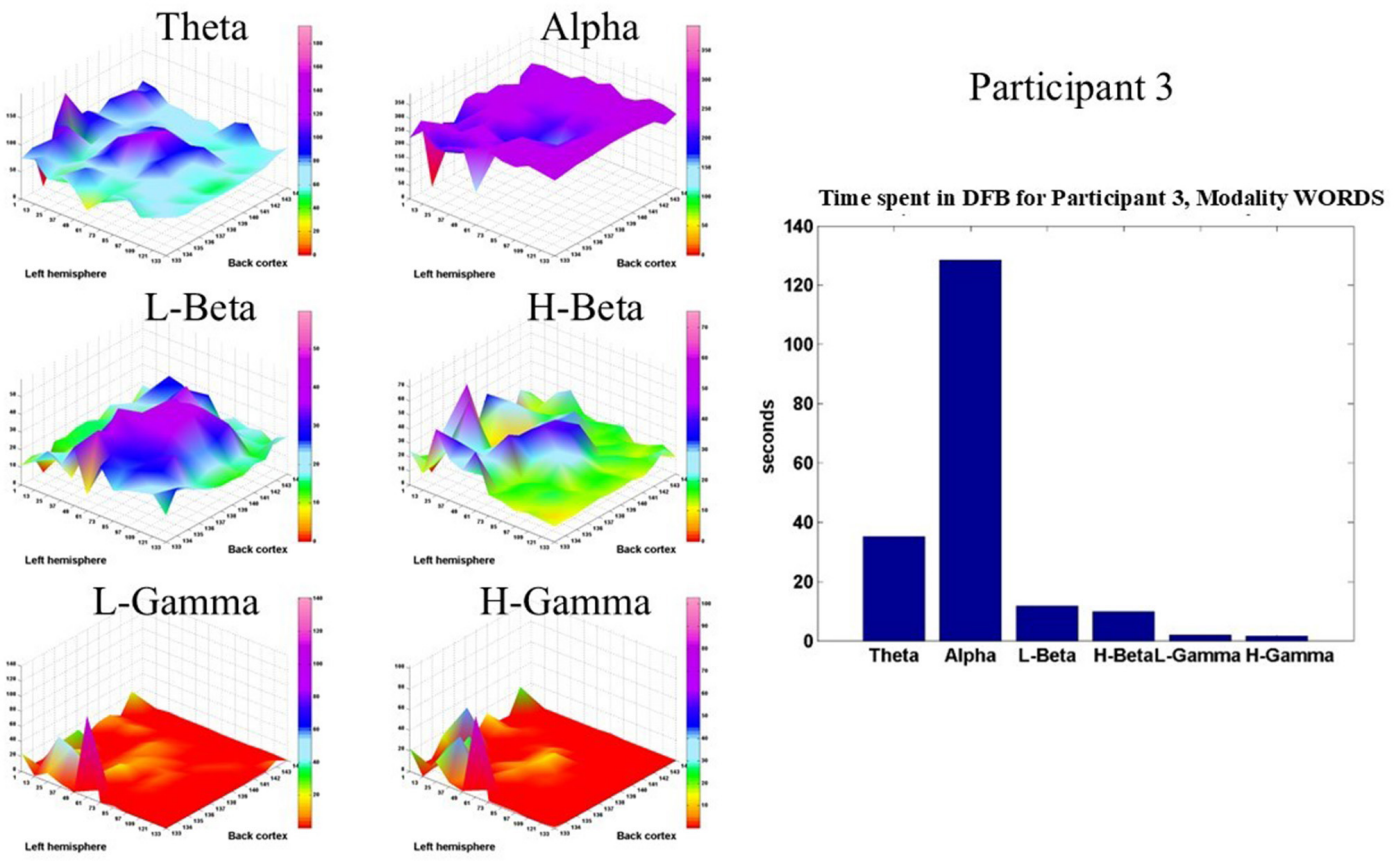
Shows, for Participant 3, in modality WORDS, six 2D spatial landscapes, in different frequency bands, of the different brain areas that measure the time that the DFB is in each band **(left)**. Overall time spent by DFB in each band **(right)**.

Figure 8 shows such displays for Participant 3 in the modality WORDS. It is clear that the Alpha band dominates in the brain dynamics of this participant. However, the theta, L-beta, and H-beta frequency bands show some significant time spent in those bands in the central and prefrontal areas.

**Figure 9:**
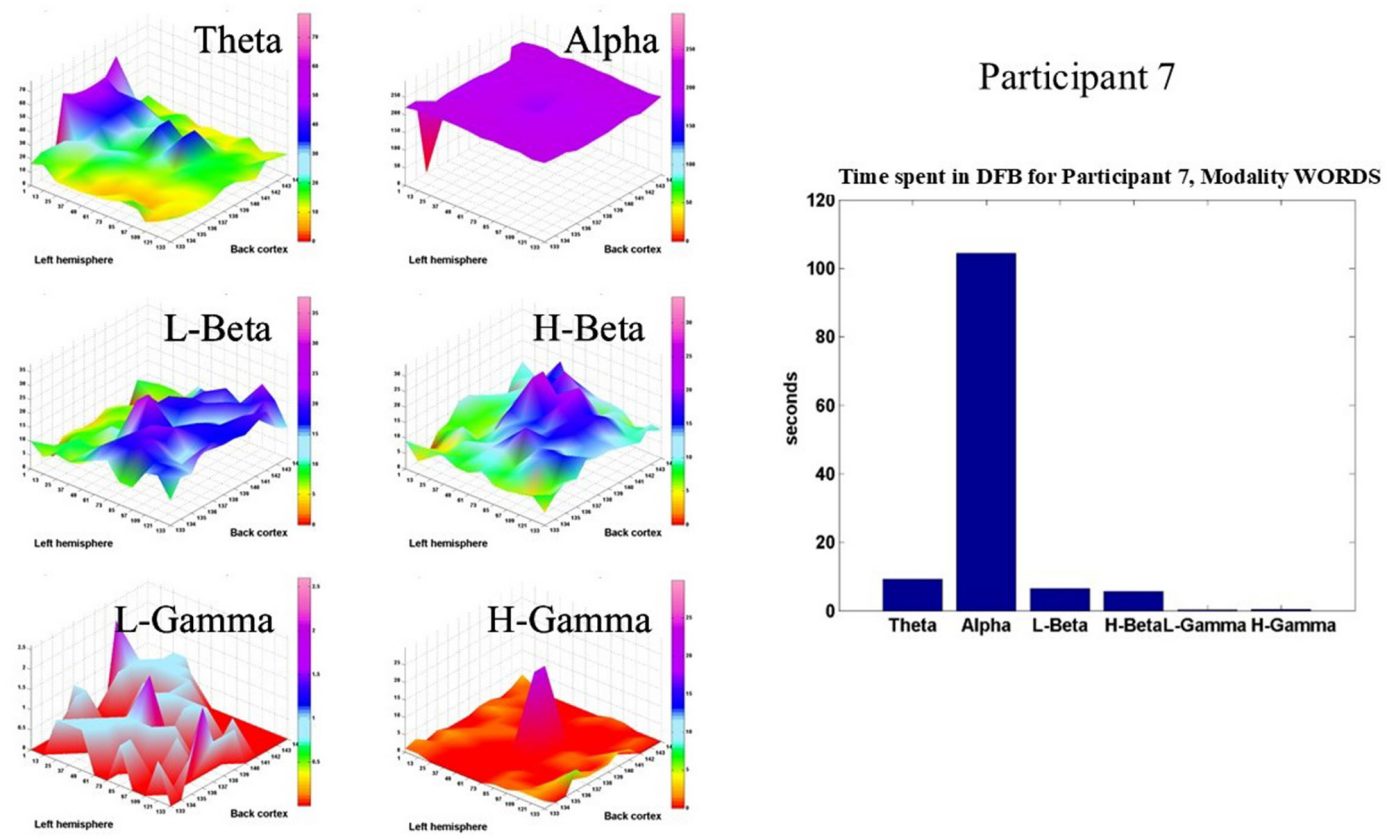
Shows, for Participant 7, in modality WORDS, six 2D spatial landscapes, in different frequency bands, of the different brain areas that measure the time that the DFB is in each band **(left)**. Overall time spent by DFB in each band **(right)**.

Figure 9 shows the same type of display for Participant 7, also in the modality WORDS. Again, the Alpha band dominates the brain dynamics of this participant. However, this participant shows a significant amount of time spent in the theta, L-beta, and H-beta frequency bands in the prefrontal, parietal, and occipital cortices, respectively.

Overall, both participants show relatively very similar behaviors. It is important to note at this stage that both participants live a peaceful life in nature and practice meditation on a regular basis. That explains why the dominant frequency band for both is the Alpha band, based on a previous recent publication (Davis et al., 2023a). Moreover, some differences are observed for both participants, as mentioned before, which is something expected since every participant's brain dynamic is very different and idiosyncratic.

This type of qualitative analysis may prove to be very useful in clinical research as a tool for diagnostic pathologies associated with certain brain patterns in certain areas and bands.

**Figure 10:**
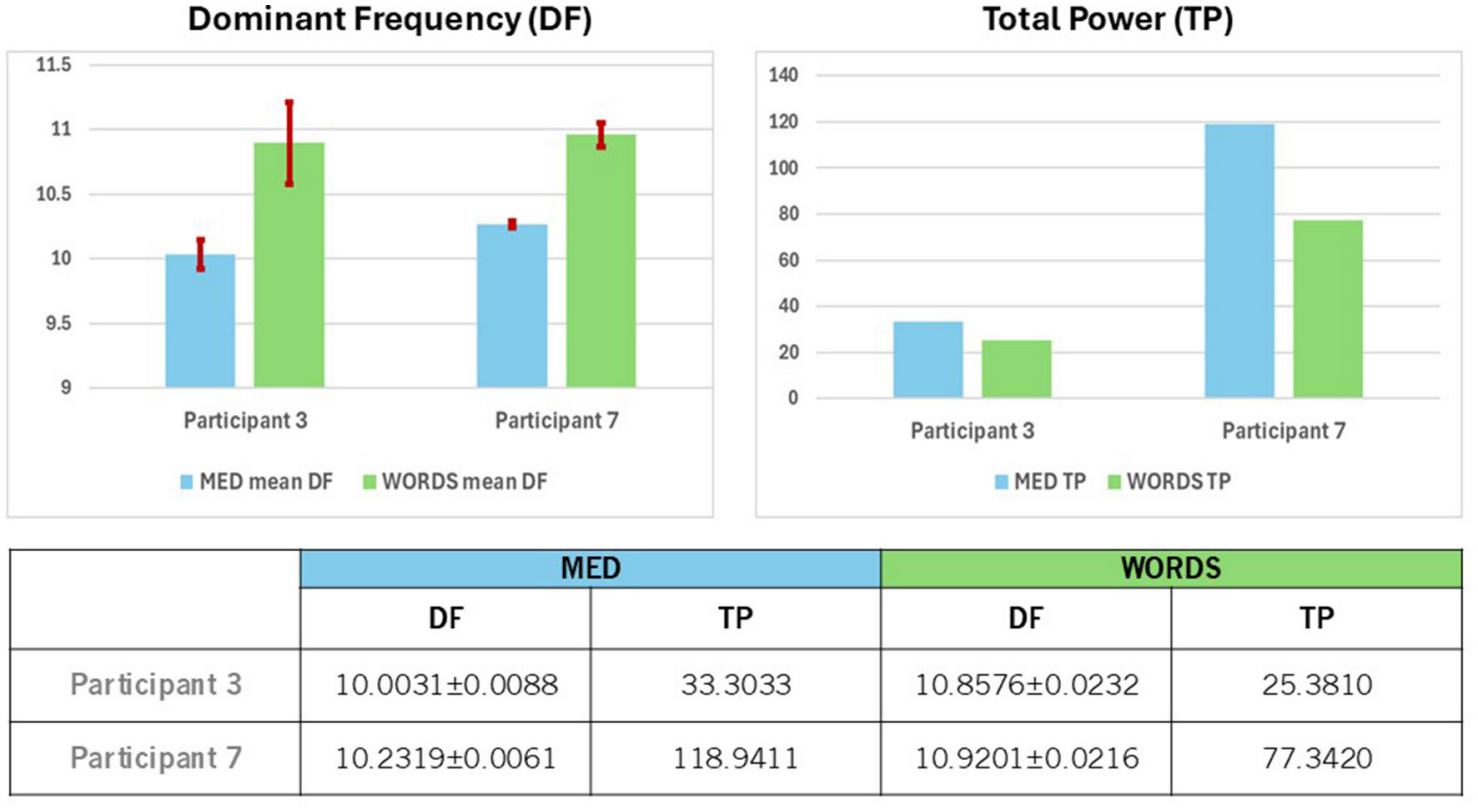
**(Top)** Shows a comparison between Participants 3 and 7 for mean values of the Dominant Frequency (DF), with 95% confidence intervals for modalities MED and WORDS **(left)** and the Total Power (TP) values **(right)**. **(Bottom)** Shows mean values of Dominant Frequency (DF) with 95% confidence intervals and the values for Total Power (TP) for modalities MED and WORDS for Participants 3 and 7.

In Figure 10 (Top and Bottom), the statistics associated with the DFB for each modality for each participant are shown. When comparing the behavior of both participants in both modalities based on DF, we observe that DF for modality WORDS shows larger numbers than for modality MED. Moreover, modality WORDS shows similar values for both participants, while for modality MED, Participant 7 shows slightly larger numbers than Participant 3, although both are clearly dominated by the Alpha frequency band.

Furthermore, in Figure 10, different behaviors based on TP for each modality for each participant are observed. The behavior of both participants in both modalities based on TP shows that TP values are smaller for modality WORDS than for modality MED.

Participant 7 displays significantly greater values for TP for both modalities. The question that arises is what that would mean when distinguishing the kind of brain dynamics that both participants show since they are of a similar age range, cultural upbringings, and lifestyle; however, one is a female and the other a male, both living in a peaceful and caring community. This is reminiscent of Freeman (1995). Such situations have been shown to promote correlations between participants' physiological rhythms, such as in Heart Rate Variability studies, that presumably, and most likely, are associated with brain dynamics, whereas Alpha brain rhythms are, in turn, associated with psychophysiological coherent states, that may be prevalent among such community members (Plonka et al., 2024). Could it be that Participants 3 and 7 are somehow synchronized in Alpha rhythms, considering that most of the time, their brain dynamics are dominated by the Alpha frequency band? This certainly needs further investigation with a larger database.

In Figure 10 (Bottom), the quantitative statistical analysis associated with the above qualitative analysis is presented.

**Figure 11:**
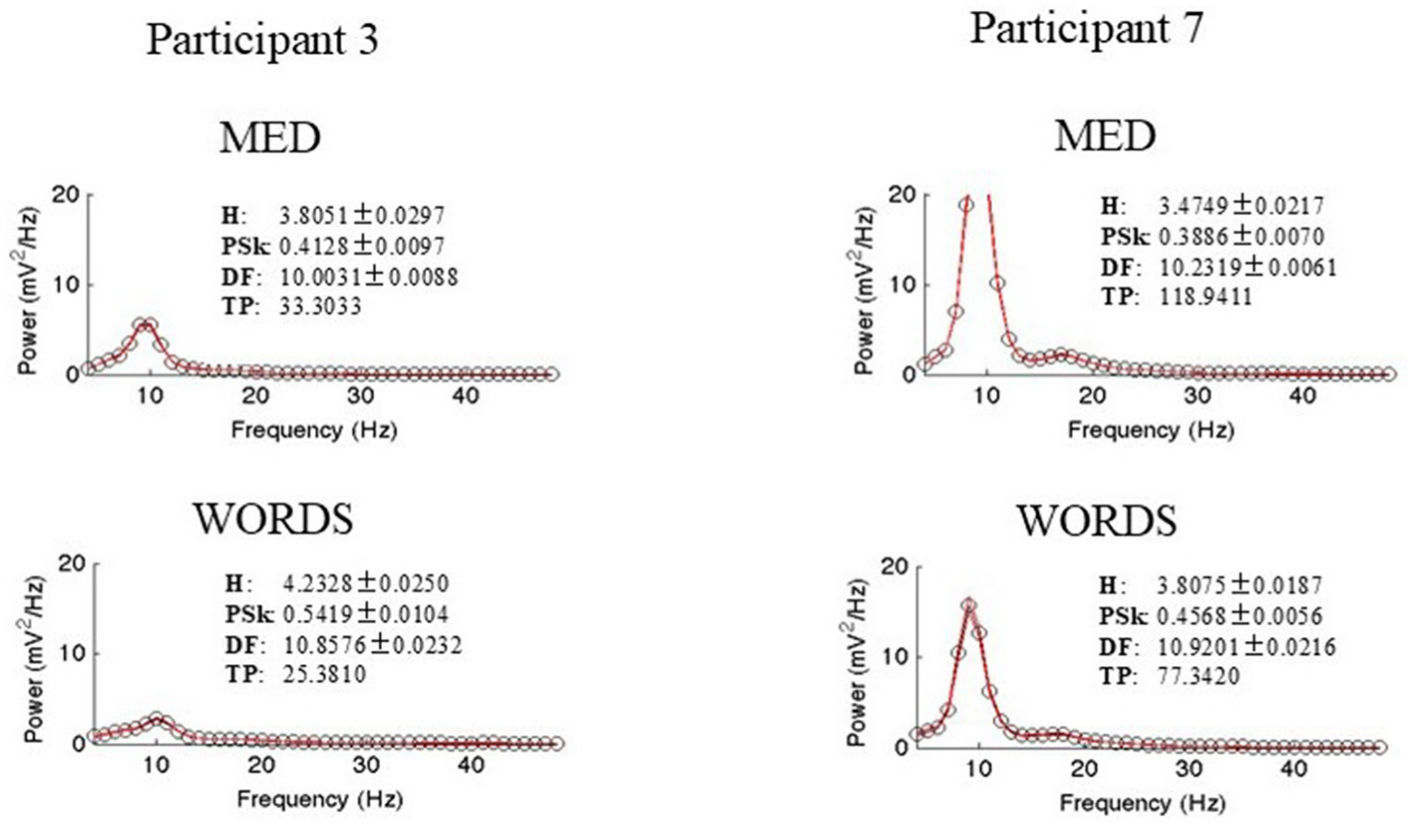
Shows power spectrum with measures of interest, namely, H, PSk, DF, and TP for both participants in both modalities.

To finalize this section, a graphic synthesis of the analysis results that are derived from the power spectrum for both participants in both modalities are presented, to obtain all the measures of interest, namely H, PSk, DF, and TP, as shown in Figure 11.

There are significant differences between the power spectrums of both participants as expected from our previous analysis. The power spectrum of Participant 7 shows a bimodal distribution, where the Alpha and Beta bands show more power than Participant 3. Moreover, modality MED shows more power for both participants. All the values for the measures of interest and plots are shown.

## 6 Some reflections on the cycle of creation of knowledge and meaning

Through the years, from 2011 to 2025, in Davis's collaboration with Walter J. Freeman, while he was alive, together with his collaboration with Robert Kozma, Grant Gillet, and Florian Schübeler, till the present day, the hypothesized cycle of creation of knowledge and meaning in different cognitive states remains his main field of study in Cognitive Science.

Davis's reflections on different categories of meanings and values (Davis, 2009) and the relevance that they have for human intentional action and values-based decision-making, points in the direction of “[…] fundamentally richer understandings that include the primacy of action, intention, emotion, culture, real-time constraints, real-world opportunities, and the peculiarities of living bodies.” (Freeman and Núñez, 1999, p. ix). This remains one of the most important areas of knowledge to develop if we ought to survive long enough to preserve humanity and advance to better scenarios of individual inner peace and social harmony, particularly in this age of AI.

The main research questions are: (a) How does the brain participate in the creation of knowledge and meaning? (b) How do we classify different cognitive states and categories of meanings when different brain oscillations are manifested in different areas of the brain, when the human being is presented with different kinds of stimuli coming from his or her environment, as well as internal signals, such as the ones generated when the human being is thinking, meditating, or being creative? Moreover, (c) What would it mean for human beings to master, at will, the creation of meanings and values of his or her choice, together with the choosing of lifestyles conducive to a sound spiritual and intellectual foundation, general wellbeing, and the power to contribute to social harmony via new social contracts?

As explained in one of the sections above, the mathematical framework used to derive a measure for meaning creation from brain signals is the Hilbert Transform methodology, from where, after a set of steps, we derive NPS that have been associated with meaning creation moments and thought patterns in previous studies (Davis et al., 2015a,b, 2024; Joshua Davis et al., 2024).

**Figure 12:**
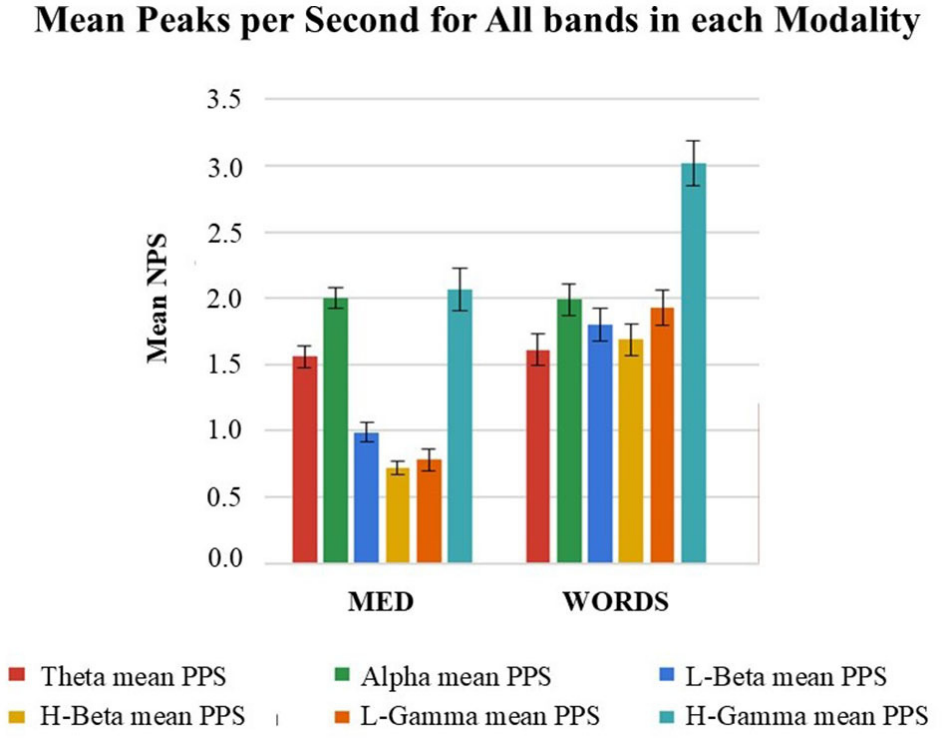
Shows mean NPS with confidence intervals for modalities MED and WORDS, in every band, for all twenty (20) participants taken together. See more general results in Davis et al. (2024).

In Figure 12, it can be observed that the Mean NPS shows significantly different behaviors for different bands within each modality and that modality MED displays much lower values than modality WORDS for the Beta and Gamma bands, while very similar values for theta and alpha. This may respond to findings associating theta and alpha as gating bands on which Beta and Gamma ride. This has been shown to relate to thalamo-cortical theta rhythms by Werbos and Davis (2017) and in studies showing the alpha and theta linkage (Freeman and Quiroga, 2013; Jensen and Mazaheri, 2010). Note that theta and alpha have also been shown to be prevalent in meditative and relaxation cognitive states (Lagopoulos et al., 2009).

**Figure 13:**
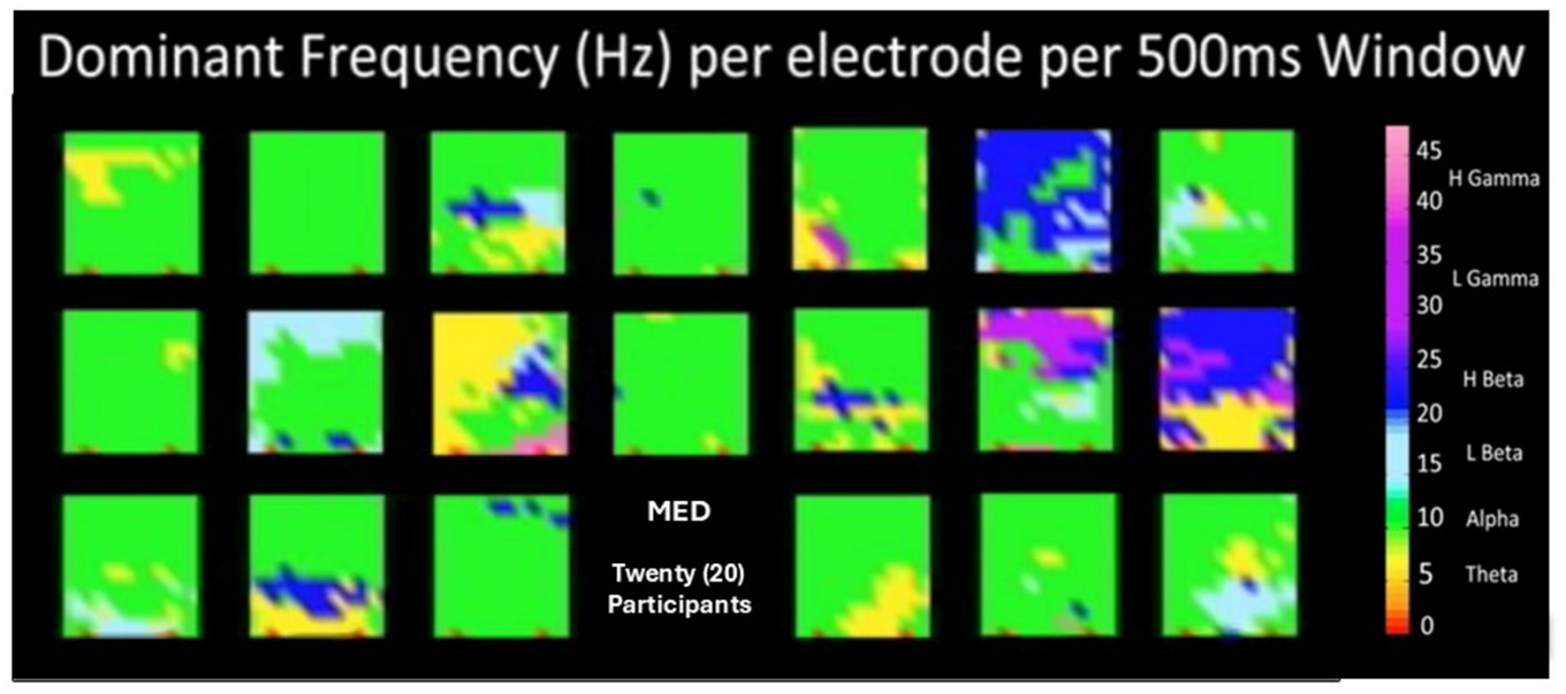
Shows a snapshot of the twenty (20) participants in modality MED. These snapshots are obtained each for every 500 ms, and when played one after the other, it creates an animation or movie. This is called the art of encephalography.

Finally, it is important to say that as part of the methodology, a set of brain dynamics movies have been derived that allow the display of spatio-temporal patterns of behavior in the measure or index of our choice. In Figure 13, a snapshot of the 20 participants in modality MED is shown. These snapshots are obtained each 500 ms, and when played one after the other, it creates an animation or movie. This is called the art of encephalography (Davis et al., 2015a,b; Davis, 2017).

These movies allow encephalographers, neuroscientists, cognitive scientists, and students to learn about brain dynamics in different cognitive states and significant moments of meaning and knowledge creation when choosing the appropriate measures and indices. As a wine *connoisseur* is trained for years to recognize wine characteristics when tasting them, an *encephalographer-connoisseur* could be trained via the art and science of brain dynamics movie watching to identify cognitive states and relevant brain events. This, when done in collaboration with systems neuroscientists and cognitive neuroscientists, can lead to the refinement of actual theories and the creation of new brain theories.

## 7 Conclusion

Freeman Neurodynamics, and part of his legacy, has been briefly introduced. He has provided the foundations for a methodology that has been presented and that incorporates the application of the Hilbert and the Fast Fourier transforms algorithms in the computation of pragmatic information indices leading to the NPS index, and Power Spectrum based indices, such as H, PSk, DF, and TP, which show significant brain events and different cognitive states. Some results have been derived from real human data to illustrate the power and benefits associated with the methodology presented. It has been pointed out briefly the implications of using this methodology in these kinds of studies for the understanding of meditative states in contrast with more engaged states, presumably accompanied by a sense of individual inner peace and general wellbeing.

We must keep in mind the limitations of the methodologies presented when applying the pragmatic information index in dealing with a complex system such as the brain, particularly when making an attempt to understand how the brain participates in the creation of meaning.

Other methodologies to deal with these complexities have been applied and also deserve careful attention (Buzsaki, 2019; Kelso and Tognoli, 2007; Zhang et al., 2019).

In general, we ought to account for individual variability, which could make it difficult to derive generalized results. One way to deal with such a challenge would be to use machine learning methods for individualized analysis considering participant's specific characteristics, particularly dealing with cognitive states characterization (Lombardi et al., 2025). This could complement and enhance the analysis. Other methods could be useful for the task, as mentioned before and shown in Werbos and Davis (2017), and also in the application of fuzzy systems to EEG signal characterization (Yu et al., 2025; Cao et al., 2020). Other methods that have been used in different fields may also apply to EEG signal analysis and deserve consideration (Versaci and Morabito, 2018; Versaci et al., 2025).

It is anticipated that the use of this general methodology will contribute to deepen our understanding of brain dynamics, diverse cognitive states, and their neuro-energetics (Noack et al., 2017); in the context of intentional action, this will certainly require more research, with a more diverse and larger database of participants to reach more conclusive results.

We dedicate this study to the memory of Walter J. Freeman, a beloved friend and collaborator to two of the authors.

## Data Availability

The data analyzed in this study is subject to the following licenses/restrictions: available on request. Requests to access these datasets should be directed to joshua.davis@auckland.ac.nz.

## Footnotes

^1^Meditation (MED), scrambled words resolution (WORDS), ambiguous images discovery (IMG), mental arithmetic (MM), listening to and differentiating between pleasant sentences and gibberish (SENT), and watching a video that displays ambiguous images with a music audio (VDO).

^2^The symbol 〈〉 represents the spatial ensemble average, where A(t), the analytic amplitude is a measure of distance that can be based on analytic amplitude AA(t), or on analytic phase AP(t). Here we have defined A(t) and *D*_e_(t) as AA(t) and ED(t), respectively.

^3^It is important to note that the measures of interest, H, PSk, TP, and DFB, are computed in windows of 500 ms. This window may vary from study to study, and it is to be determined by the research team according to the specific needs for analysis. Moreover, DFB and DF will be used interchangeably unless noted.
